# Would raising the total cholesterol diagnostic cut-off from 7.5 mmol/L to 9.3 mmol/L improve detection rate of patients with monogenic familial hypercholesterolaemia?

**DOI:** 10.1016/j.atherosclerosis.2015.01.028

**Published:** 2015-04

**Authors:** M. Futema, M. Kumari, C. Boustred, M. Kivimaki, S.E. Humphries

**Affiliations:** a Centre for Cardiovascular Genetics, British Heart Foundation Laboratories, Institute of Cardiovascular Science, The Rayne Building, University College London, London, WC1E 6JF, UK; b University of Essex, Wivenhoe Park, Colchester, Essex, CO4 3SQ, UK; c NE Thames Regional Genetics Service, Great Ormond Street Hospital for Children, London, UK; d Department of Epidemiology & Public Health, UCL Institute of Epidemiology & Health Care, University College London, London, UK

**Keywords:** Familial hypercholesterolaemia, Diagnosis, *LDLR*, *APOB*, Cholesterol

## Abstract

A previous report suggested that 88% of individuals in the general population with total cholesterol (TC) > 9.3 mmol/L have familial hypercholesterolaemia (FH). We tested this hypothesis in a cohort of 4896 UK civil servants, mean (SD) age 44 (±6) years, using next generation sequencing to achieve a comprehensive genetic diagnosis. 25 (0.5%) participants (mean age 49.2 years) had baseline TC > 9.3 mmol/L, and overall we found an FH-causing mutation in the *LDLR* gene in seven (28%) subjects. The detection rate increased to 39% by excluding eight participants with triglyceride levels over 2.3 mmol/L, and reached 75% in those with TC > 10.4 mmol/L. By extrapolation, the detection rate would be ∼25% by including all participants with TC > 8.6 mmol/L (2.5 standard deviations from the mean). Based on the 1/500 FH frequency, 30% of all FH-cases in this cohort would be missed using the 9.3 mmol/L cut-off. Given that an overall detection rate of 25% is considered economically acceptable, these data suggest that a diagnostic TC cut-off of 8.6 mmol/L, rather than 9.3 mmol/L would be clinically useful for FH in the general population.

## Highlights

• 28% of sequenced UK individuals with total cholesterol >9.3 mmol/L were found to have an FH mutation using NGS.
• Detection rate was higher (39%) in individuals with triglycerides lower than 2.3 mmol/L.
• By extrapolation, a 8.6 mmol/L (2.5 SD from the mean) cholesterol cut-off may be most economically sustainable.

## 1 Introduction

Familial Hypercholesterolaemia (FH) in its classical form is an autosomal dominant disorder, characterised by increased plasma levels of low-density-lipoprotein-cholesterol (LDL-C) and total cholesterol (TC) from birth and premature cardiovascular events. FH affects about 1/200 [1] to 1/500 [2] individuals of the Caucasian population, with an estimated 1.8 to 4.5 million people affected in Europe [3], and between 120,000–240,000 people in the UK with heterozygous FH, of whom at least 75% are undiagnosed [4]. The clinical diagnosis in the UK is based on the criteria of elevated TC and LDL-C levels (>7.5 mmol/L and >4.9 mmol/L, respectively), and family history of early coronary heart disease (CHD) and/or elevated cholesterol levels, when patients are given a diagnosis of possible FH (PFH), and the presence of clinical features such as tendon xanthomas, when patients are given a diagnosis of definite FH (DFH) [2]. In Europe, a scoring system developed in Holland [3] is more widely used.

Recently, the need for implementation of universal or targeted screening for FH to tackle the disease underdiagnosis and undertreatment has been highlighted [1,3]. Early identification of at-risk individuals allows changes in lifestyle including dietary intervention, and drug treatment, which have been shown to reduce coronary atherosclerosis and to improve life expectancy [5–7]. Cascade testing using the family mutation to identify carrier relatives unambiguously is a cost-effective method of finding additional FH patients, and has been used extensively in other countries in Europe, most notably in Holland, for the last five years [8]. Improved identification of FH cases has recently been included in the new UK Department of Health Cardiovascular Outcomes Strategy (https://www.gov.uk/government/publications/improving-cardiovascular-disease-outcomes-strategy), and a newly issued set of NICE Quality Standards (QS41) underline the existing guidance for the diagnosis, cascade screening and management of FH in England [9]. Based on the Simon Broome criteria, individuals with TC > 7.5 mmol/L and/or LDL-C >4.9 mmol/L should be assessed for a clinical diagnosis of FH. However, there is concern that this proposed cut-off is too low and will result in many false positive diagnoses and a high workload for lipid clinics.

## 2 Hypothesis and methods

A previous study [10] suggested that 88% of the general population in the US (age >40 years) with TC > 9.3 mmol/L (and/or LDL-C >6.8 mmol/L) and normal triglycerides (TG < 2.3 mmol/L) are expected to have an FH-causing mutation. We aimed to test this hypothesis using targeted next generation sequencing (NGS) (Illumina TruSeq Custom Amplicon and MiSeq Illumina sequencer) methods for genetic diagnosis of FH [11] in the Whitehall II prospective cohort study (WHII) of British civil servants [12]. The criteria of our standard variant calling pipeline were: coverage ≥30×, minimum of five reads for an altered allele, Phred quality ≥20, and a strand bias filter. To ensure that variants were not missed a *sensitive* pipeline was used (coverage ≥15×, minimum of two reads for an altered allele, Phred quality ≥ zero, no strand bias filter). Copy number variants (CNVs) were called using the ExomeDepth package [13]. All variants were confirmed by Sanger sequencing or for CNVs by MLPA [14]. The polygenic cause of hypercholesterolaemia in the WHII cohort was also assessed using a 6-SNP genetic risk score [15,16].

## 3 Results

**Fig. 1:**
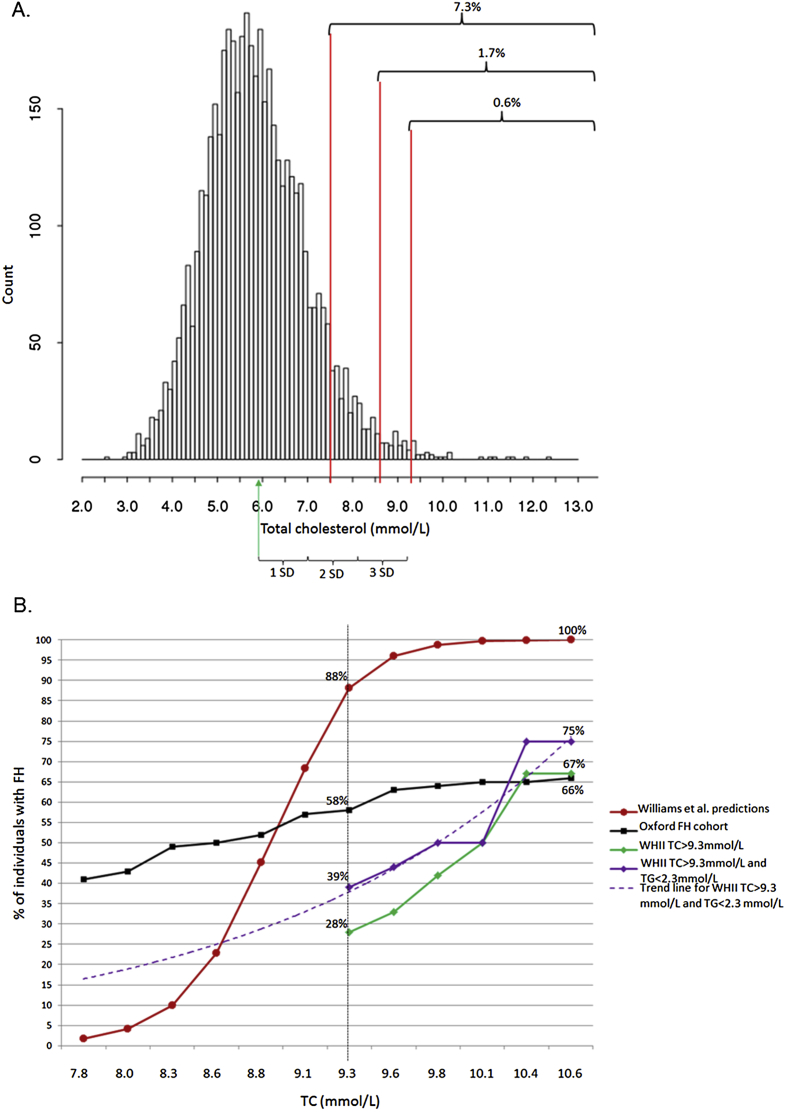
The relationship of TC and FH mutation detection rate. A. TC distribution in WHII (n = 4896). Red lines indicate the proposed TC cut-offs (7.5 mmol/L, 8.6 mmol/L and 9.3 mmol/L) and the proportion of the cohort above those cut-offs. Green arrow indicates the TC mean (5.9 mmol/L). One, two and three standard deviations (SD) to the right of the mean are also marked. B. Percentage of adults with FH predicted by Williams et al. in comparison to FH mutation carriers in WHII and Oxford FH [18]. (For interpretation of the references to colour in this figure legend, the reader is referred to the web version of this article.)

In the cohort of 4896 WHII participants recruited in 1985–99 (baseline characteristics shown in the Supplement Table S1), for whom DNA samples were available, we identified 25 subjects (0.5%) with a baseline TC > 9.3 mmol/L (TC distribution shown in Fig. 1A). This group was sequenced for mutations in four FH genes (*LDLR*, *APOB*, *PCSK9* and *LDLRAP1*) and genotyped for six LDL-C-associated SNPs (rs629301 in *CELSR2*, rs1367117 in *APOB*, rs6544713 in *ABCG5/8*, rs6511720 in *LDLR*, and rs429358 and rs7412 in *APOE*). Causality of identified variants was thoroughly assessed considering previously published studies and *in silico* tools [17].

Considering the conservative 1/500 frequency of FH, we would expect 10 FH individuals in this cohort. An *LDLR* mutation, including one CNV (Supplement Fig. S1) was found in 28% (n = 7) of sequenced individuals (Table 1), which accounted for 70% of the estimated FH in WHII. The sensitive variant-calling pipeline did not detect any additional mutations. The *APOB* p.R3527Q mutation, which is known to account for about 5% of FH mutations in the UK [18] was not found. However, our previous analysis of the WHII cohort using the Metabochip [19] identified two carriers of the p.R3527Q mutation. FH patients affected by the *APOB* mutation are known to have lower TC/LDL-C than those with a defective LDL-receptor [18,20]. In this case the two p.R3527Q mutation carriers had TC of 9.3 mmol/L and 5.9 mmol/L and therefore they were missed by the >9.3 mmol/L cholesterol cut-off criteria.

Polygenic hypercholesterolaemia is thought to account for the majority of clinically diagnosed FH cases where no mutation can be found [15,21]. Out of the 18 mutation negative individuals eight had a 6-SNP LDL-C genetic risk score above the top quartile of the score distribution, and 16 had a score in the top three quartiles of the score, which is associated with a >95% likelihood of polygenic cause of hypercholesterolaemia [16]. Using the genetic information, we were therefore able to identify the likely genetic cause of their high TC in 92% of sequenced individuals (seven monogenic and 16 polygenic). Of the unexplained two individuals, one had high TG level (2.75 mmol/L), which could suggest a diagnosis of familial combined hypercholesterolaemia. The polygenic cause of hypercholesterolaemia in these patients should be considered when designing a screening protocol for the proband's relatives, since the efficiency of cascade testing in polygenic hypercholesterolaemia patients is likely to be compromised in comparison to patients with monogenic FH [15,16].

Mutation carriers in the studied cohort had significantly higher TC and significantly lower TG levels than non-mutation carriers (Table 2), which confirms our previous findings that the FH mutation detection rate correlates positively with pre-treatment TC and negatively with pre-treatment TG [18].

We repeated the analysis after excluding individuals with TG > 2.3 mmol/L (n = 8, all were mutation negative), which led to an increase in the percentage of subjects with TC > 9.3 mmol/L who were FH-mutation carriers from 28% to 39% (Fig. 1B). We then compared these results with a previously published cohort of FH patients from an Oxford lipid clinic, who had been identified using the standard Simon Broome criteria of TC > 7.5 mmol/L plus having a family history of high cholesterol or premature CHD [2]. In this sample of DFH and PFH patients overall the mutation the percentage of FH mutation carriers was 37% and among individuals with TC > 9.3 mmol/L was 58%, which was significantly higher than in the WHII subjects (*p* = 0.01 (χ^2^)) (Fig. 1B), reinforcing the utility of family history information in identifying mutation carriers.

## 4 Discussion

Based on the classical frequency of heterozygous FH of 1/500 we expected to find 10 mutation carriers in the ∼5000 subjects included here, and successfully identified seven i.e. 70% of predicted. However, if the true frequency of FH in the UK is similar to the 1/250 reported in Denmark [1], with this estimate supported by the frequency of 1/217 of *LDLR* mutation carriers observed by exome sequencing [22], our overall detection rate would be only 35% (i.e. 7 of the 20 expected in the 5000 subjects examined). While it is possible that some mutation carriers were missed for technical reasons in the next generation sequencing or bioinformatics methods used, reducing the stringency of calling did not identify any additional variants, which were confirmed by Sanger sequencing. It is most likely that some FH patients carrying “mild” mutations (or with few common LDL-C raising variants i.e. a low 6-SNP score) would not have been included in the selected 25 subjects, as clearly shown by the exclusion of the two previously identified *APOB* p.R3527Q carriers. For example, in the study of 101 mutation-positive FH patients from the Oxford lipid clinic [18] mean TC in *LDLR* mutation carriers was 9.81 (±1.52) mmol/L, and in the *APOB* p.R3527Q carriers was 9.12 (±0.85) mmol/L. In the Oxford group, only 58% of mutation carriers would have been detected using the 9.3 mmol/L TC threshold proposed by Williams et al. [9].

The reason for the significantly lower detection rate at a TC cut-off of 9.3 mmol/L in WHII compared to that predicted by Williams et al. (28% vs 88%) is unclear, but will be influenced by the distribution of TC levels in the two samples (Utah mean TC = 5.3 ± 1.0 mmol/L vs. 5.9 ± 1.1 mmol/L in WHII), as well as different underlying genetic and environmental features of the two groups. While we do not have data on the number of FH-causing mutation carriers that would have been detected if sampling had been carried out at lower TC levels, extrapolation of the detection rate data in Fig. 1 in the WHII sample where subjects with TG > 2.3 mmol/L were excluded, suggests that roughly 15% of subjects with TC > 7.5 mmol/L would be carriers. If an overall detection rate of 25% were considered economically acceptable (close to the reported detection rate in *BRACA1/2* in women with a family history of breast cancer [23] then a TC cut-off of >8.6 mmol/L in 50 year old participants may be clinically useful. Although in this study we were unable to assess the percentage of FH mutation carriers in individuals with TC in the range of 8.6–9.3 mmol/L, we estimated that using a cut-off of 2.5 standard deviations from the TC mean of a screened population would achieve a ∼25% mutation detection rate (Fig. 1A). Based on the WHII cohort, lowering the TC cut-off to 8.6 mmol/L would increase the number of individuals who need the FH genetic test by three times (from 0.5% to 1.7% of the population), and by 10 times if the Simon Broome FH cut-off was used.

Taken together, in a general population opportunistic screening situation, these findings may help to select individuals for an FH DNA test using the TC measurement. However, the additional assessment of family history and TG levels in potentially affected individuals will significantly improve the detection rate.

## 5 Limitations

Although we have previously demonstrated that NGS methods for the detection of FH mutations are robust and more sensitive than standard methods, including Sanger sequencing [24], we recognise that Sanger re-sequencing of the FH genes in our cohort could provide additional evidence that no mutations were missed. The 8.6 mmol/L TC cut-off was identified using an extrapolation approach, which is subject to uncertainty. Lack of sequencing data for individuals with TC ≤ 9.3 mmol/L prevents the analysis of the true sensitivity/specificity of the FH diagnosis in these individuals.

## Figures and Tables

**Table 1:** *LDLR* mutations identified in the Whitehall II cohort. All variants were in a heterozygous state and were confirmed by Sanger sequencing or MLPA for CNVs.

Number of carriers	DNA change^a^	Protein change	Baseline total cholesterol (mmol/L)
1	c.266G > A	p.(Cys89Tyr)	11.6
1	c.1048C > T	p.(Arg350*)	12.4
1	c.1135T > C	p.(Cys379Arg)	10.2
1	c.1238C > T	p.(Thr413Met)	9.8
1	c.1444G > A	p.(Asp482Asn)	9.4
1	c.1845 + 11C > G	*Splicing changed*	11.2
1	c.68−?_940+?del	*Deletion of exons 2 to6*	11.9

a Sequence number using *LDLR* transcript: NM_000527.4 (numbered from ‘A’ (no.1) in the ‘ATG’ codon). Sequence density plots were used to determine the presence of insertions and deletions and are presented in Supplementary material.

**Table 2:** Baseline characteristics (Mean ± SD) of the mutation positive and negative subjects.

	Mutation +ve (n = 7)	Mutation −ve (n = 18)	*p*
Age (years)	49.1 (±6)	49.2 (±5)	0.5
6-SNP LDL-C genetic score	0.67 (±0.1)	0.71 (±0.3)	0.3
Baseline TC (mmol/L)	10.9 (±1.1)	9.8 (±0.5)	0.007
Baseline TG (mmol/L)	1.3 (±0.7)	2.5 (±0.8)	0.004
Number of treated individuals (%)	6 (86)	9 (50)	0.08
Post-treatment TC (mmol/L)	5.6 (±0.3)	5.7 (±1.1)	0.5
TC reduction after treatment (%)	49 (±5)	41 (±12)	0.05

LDL-C, low-density-lipoprotein cholesterol; SNP, single nucleotide polymorphism; +ve, positive; −ve, negative. Lipids concentrations were not normally distributed, and are presented as geometric means with an approximate standard deviation in brackets.
