# Supplementary material for: Would raising the total cholesterol diagnostic cut-off from 7.5 mmol/L to 9.3 mmol/L improve detection rate of patients with monogenic familial hypercholesterolaemia?
Source: Atherosclerosis. 2015 Apr;239(2):295–8. doi: 10.1016/j.atherosclerosis.2015.01.028 (PMC4373788; doi:10.1016/j.atherosclerosis.2015.01.028)

**SUPPLEMENTARY DATA**

**‘Would raising the total cholesterol diagnostic cut-off from 7.5mmol/L to 9.3mmol/L improve detection rate of patients with monogenic Familial Hypercholesterolaemia?’**

Futema M^1^, Kumari M^2^, Boustred C^3^, Kivimaki M^4^, and Humphries SE^1^

**Table S1.**

Baseline characteristics of the Whitehall II cohort (n=4896). TG and HDL-C measurements were available for 845 subjects.

|  | **%age Male** | **AGE (years)** | **TC (mmol/L)** | **TG (mmol/L)** | **HDL-C (mmol/L)** | **BMI** |
| --- | --- | --- | --- | --- | --- | --- |
| **Mean (SD)** | 75 | 44 (±6) | 5.9 (±1.1) | 2.1 (±1.4) | 1.47 (±0.4) | 24 (±3.3) |

^SD = standard deviation^

**Figure S1.**

Copy Number Variant (CNV) c.68-?_940+?del (deletion of exons 2 to 6) in the *LDLR* gene. A. ExomeDepth results indicating deletion of exons 3 to 6. The crosses show the ratio of observed/expected number of reads for the test sample. The grey shaded region shows the estimated 99% CI for this observed ratio in the absence of CNV call. The presence of contiguous exons with read count ratio located outside of the CI is indicative of a heterozygous deletion or duplication in a sample. B. MLPA results indicating a deletion of exons 2 to 6. MLPA test was taken as the gold standard.

A.


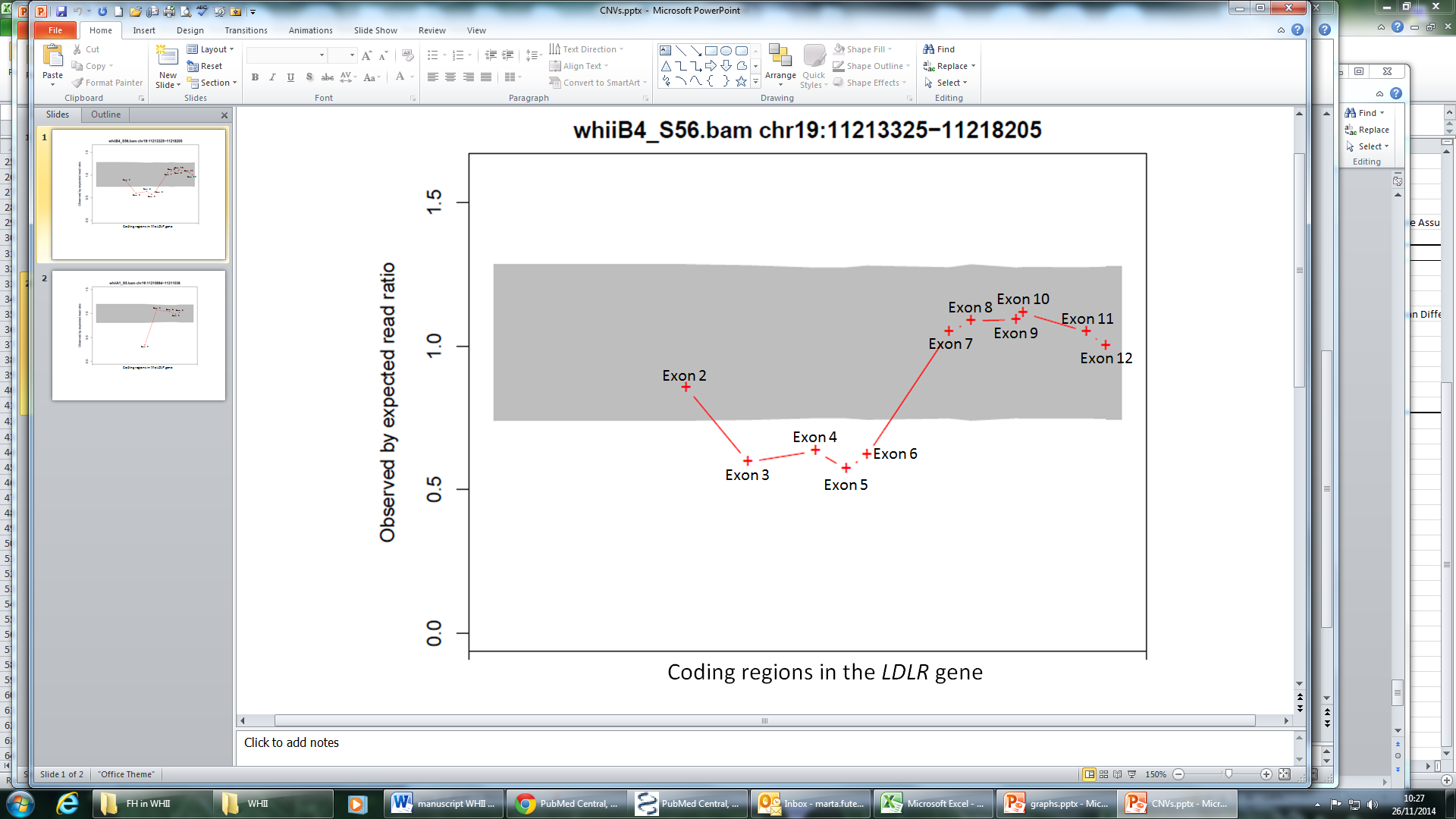


B.


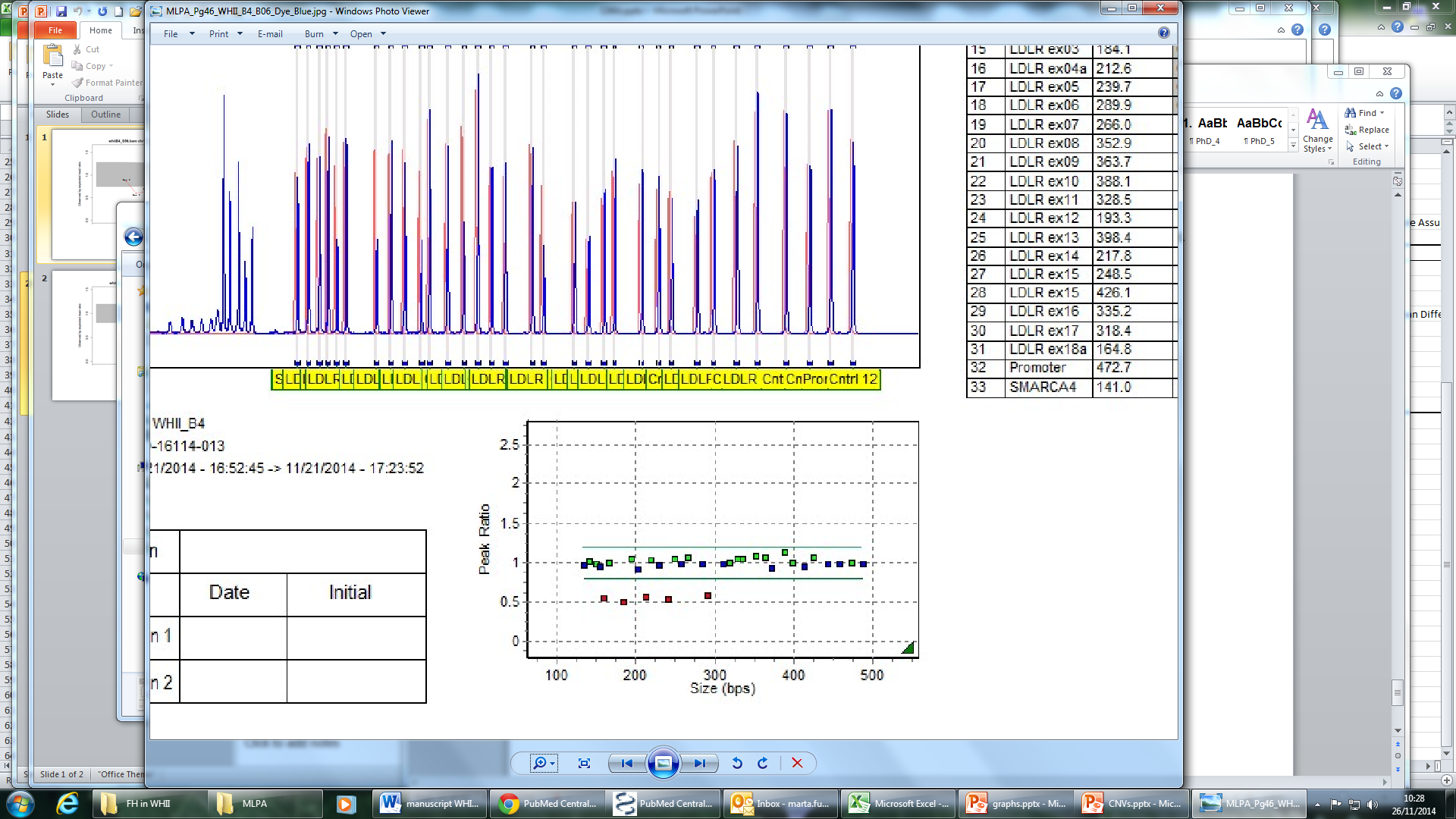

Supplement: Supplementary file 1 [file mmc1.docx]
